# Pseudorabies Virus ICP0 Abolishes Tumor Necrosis Factor Alpha-Induced NF-κB Activation by Degrading P65

**DOI:** 10.3390/v14050954

**Published:** 2022-05-02

**Authors:** Xiangbo Zhang, Jingying Xie, Ming Gao, Zhenfang Yan, Lei Chen, Suocheng Wei, Ruofei Feng

**Affiliations:** 1 Key Laboratory of Biotechnology and Bioengineering of State Ethnic Affairs Commission, Biomedical Research Center, Northwest Minzu University, Lanzhou 730030, China; zhangxiangbowork@163.com (X.Z.); xjy_1314@126.com (J.X.); yzhenfang@163.com (Z.Y.); chenleiwork7@163.com (L.C.); 2 College of Life Science and Engineering, Northwest Minzu University, Lanzhou 730030, China; gaoming001222@163.com; 3 Gansu Tech Innovation Center of Animal Cell, Biomedical Research Center, Northwest Minzu University, Lanzhou 730030, China

**Keywords:** pseudorabies virus, ICP0 protein, P65, NF-κB signaling pathway

## Abstract

Nuclear factor κB (NF-κB) is involved in a wide range of innate immune activities in host cells and serves as an important component of a host’s immunity system. To survive in infected cells, viruses have evolved intricate strategies to evade the host immune response. Pseudorabies virus (PRV) is a member of the alpha herpesvirus family and is capable of causing reproductive and neurological dysfunction in pigs. PRV has a large DNA genome and therefore has the ability to encode numerous proteins that modulate host innate immune responses. In the present study, we demonstrated that the PRV-encoded immediate early protein ICP0 inhibits the tumor necrosis factor alpha (TNF-α)-mediated NF-κB signaling pathway. An in-depth study showed that ICP0 protein was able to limit NF-κB activation and decreased the expression of inflammatory cytokines interleukin-6 (IL-6) and interleukin 8 (IL-8). In addition, ICP0 blocked the activation of NF-κB through interacting with p65, degrading its protein expression and limiting its phosphorylation. PRV protein ICP0 is shown for the first time to enable escape from innate immune response through the regulation of NF-κB during PRV infection. These results illustrate that PRV ICP0 is able to block NF-κB activation. This mechanism may represent a critical role in the early events leading to PRV infection.

## 1. Introduction

Nuclear factor-κB, also known as NF-κB, is an important component of intrinsic immunity in the body [1,2,3]. Activation of the NF-κB signaling pathway can be divided into classical and non-classical pathways, with the classical pathway being mainly through the degradation of IκB proteins to allow the release of NF-κB dimers; the non-classical pathway, on the other hand, is processed by p100 and p52, allowing activation of the signaling pathway [4,5]. When cells are subjected to various extracellular stimuli, IκB kinase is activated, leading to phosphorylation of IκB protein and ubiquitination, after which the IκB protein is degraded and NF-κB dimers are released [6]. The NF-κB dimer is then further activated by various post-translational modifications and is translocated to the nucleus to participate in the transcriptional regulation of activated genes and the innate immune response. In the nucleus, it binds to the target gene to facilitate transcription of the target genes [7].

ICP0 is a ring-finger E3 ubiquitin ligase that belongs to the group of early-stage proteins encoded by alpha herpesvirus [8]. The enzyme binds directly to the component proteins of the Ub pathway to inactivate the cellular processes that underlie host immune defense and limit the progression of viral infection [9,10,11]. Many studies have shown that ICP0 plays a key role in the HSV-1 infection cycle, among many other functions, which are required to facilitate the efficient initiation of lytic infection and the activation of the regeneration of the viral genome from latency [12]. Regarding how HSV-1 counteracts the natural immune response of the host, many studies have shown that ICP0 can inhibit the activation of IFN-β and nuclear factor-κB (NF-κB) [13,14]. Although the HSV-1 ICP0 protein has multiple immune evasion mechanisms, the immune evasion function of the PRV ICP0 protein is not well understood.

Current studies on PRV proteins have focused on their antagonism to type I IFN signaling pathways, especially the cGAS-STING-mediated signaling pathway. As the DNA sensing pathway induced by innate immunity plays a critical role in controlling PRV infection, PRV proteins evolved complex mechanisms to antagonize the innate immune response. Several studies have indicated that PRV UL13 inhibits the IFN-β production by targeting IRF3 in a kinase-dependent manner [15,16,17]. PRV UL24 efficiently inhibited cGAS-STING-mediated IFN production by interacting with interferon regulatory factor 7 (IRF7) and degrading its expression through the proteasome pathway [18]. In addition, PRV gE is involved in counteracting cGAS-STING-mediated IFN production through degrading CBP [19]. Our previous study also found PRV US3 protein could inhibit cGAS-STING-mediated IFN production by interacting with IRF3 and degrading its expression [20]. There are a few studies on PRV immune evasion and the NF-κB signaling pathway. A recent study showed that PRV UL24 protein abrogated TNF-α-mediated NF-κB activation by interacting with p65 and promoting it for proteasomal degradation [21]. All in all, the study on the evasion of host immune response mediated by PRV viral proteins is very limited.

In this study, we defined the role of PRV ICP0 protein in the inhibition of NF-κB pathway activation. Our results indicated that the PRV ICP0 protein could significantly inhibit TNF-α-mediated NF-κB activation. Additionally, ICP0 prevented the degradation of IκBα and then degraded p65 to inhibit the expression of inflammatory factors IL-6 and IL-8. Co-immunoprecipitation analysis demonstrated that ICP0 interacted with p65 and degraded its expression through proteasome pathway. Meanwhile, ICP0 also inhibited the phosphorylation and nuclear translocation of p65. In conclusion, this study is the first to describe the role of ICP0 in antagonizing the NF-κB pathway. The attenuation of NF-κB activation by PRV ICP0 protein may represent an essential accommodation to enable virus persistence within the host.

## 2. Materials and Methods

### 2.1. Cells and Virus

PK15 and HEK293 cells were cultured with Dulbecco’s modified Eagle medium (DMEM, BAILING) supplemented with 10% fetal bovine serum, at 37 °C in a 5% CO_2_ incubator. PRV Bartha-61 strain was propagated in BHK-21 cells, and the supernatants of infected cells were clarified and stored at −80 °C.

### 2.2. Antibodies and Reagents

Anti-Flag tag rabbit polyclonal antibody (D191041), horseradish peroxidase (HRP)-conjugated goat anti-rabbit IgG (D110058) and HRP-conjugated goat anti-mouse IgG (D110087) were purchased from Sangon Biotech (Shanghai, China). NF-κB p65 rabbit polyclonal antibody (10745-1-AP), GAPDH mouse monoclonal antibody (60004-1-Ig), Myc tag mouse monoclonal antibody (60003-2-Ig), NFκB1 rabbit polyclonal antibody (14220-1-AP) and TAK1 rabbit polyclonal antibody (12330-2-AP) were purchased from Proteintech (Wuhan, China). Anti-HIST3H3 polyclonal antibody (K106623P) was purchased from Solarbio (Beijing, China). IκBα rabbit polyclonal antibody KO validated (AF5204), phospho-IκBα (Ser32/36) rabbit polyclonal antibody (AF5851) and phospho-NF-κB p65 (Ser276) rabbit polyclonal antibody (AF5875) were purchased from Beyotime (Shanghai, China). *TransStart* Top Green qPCR SuperMix (+Dye II) was purchased from Transgen (Beijing, China). Cell membrane/cytoplasm/nuclear membrane protein step extraction kit (BB-31042) was purchased from BestBio (Shanghai, China). Lipofectamine 3000 was purchased from Invitrogen. Chemical reagents RNase inhibitor (Thermo Fisher, Waltham, MA, USA), MG132 (Beyotime, Nantong, China), chloroquine (CQ) (tlrl-chq, InvivoGen, San Diego, CA, USA), Ac-DEVD-CHO (Beyotime) and TNF-α (InvivoGen) were purchased from indicated manufacturers.

### 2.3. Plasmids

A plasmid encoding Flag-tagged p65 was constructed by molecular cloning methods. A Myc-tagged ICP0 plasmid was constructed in-house. All plasmids were verified by sequencing. The primer sequences used in this study are available upon request.

### 2.4. Western Blotting

Cells were harvested and whole-cell extracts were prepared with lysis buffer RIPA (Solarbio, Beijing, China). Cell extracts were subjected to 10% or 15% SDS-PAGE, and the separated proteins were transferred to PVDF membranes (Millipore, Berlington, MA, USA). The PVDF membranes were incubated with specific primary and HRP-conjugated secondary antibodies. GAPDH or β-actin served as loading control. The proteins were detected using ECL Blotting Substrate (Bio-Rad, Hercules, CA, USA).

### 2.5. Co-Immunoprecipitation Assay

Cells were collected with lysis buffer supplemented with phosphatase inhibitor cocktail and incubated with anti-Flag or anti-p65 antibody for 12 h at 4 °C. Then, 10 µL of Protein G agarose slurry (Beyotime, Nantong, China) was added to each lysate. After incubation for 4 h at 4 °C, the lysates were centrifuged at 2500 rpm for 5 min. The beads were collected and washed 5 times with ice-cold PBS. The precipitates were mixed with SDS buffer and boiled for 5 min at 95 °C. After centrifugation at 6000 rpm for 1 min, the supernatant was collected and used for Western blot analysis.

### 2.6. RNA Extraction and RT-qPCR

mRNA transcription levels for NF-κB-dependent genes such as IFN-β, IL-6 and IL-8 were determined by relative quantitative PCR (RT-qPCR). Cellular RNA was isolated and reverse-transcribed to cDNA. Methods were performed as previously described [22]. Primers for RT-qPCR are available upon request.

### 2.7. Transfection

Plasmid DNA was transfected into PK15 cells using Lipofectamine 3000 (Invitrogen, Waltham, MA, USA). All experiments were conducted in accordance with the company’s instructions. Cells were then infected with PRV for 24 h at an MOI of 0.01 (except for the cases mentioned in the text) to test the effect of ICP0 on PRV replication. In co-transfection experiments, ICP0 and reporter gene constructs were used in a 1:1 mass ratio.

### 2.8. Nuclear and Cytoplasmic Extraction

PRV-infected or uninfected PK15 cells were washed in PBS with 400 μL of Extract A, 2 μL of protease inhibitor and 2 μL of phosphatase inhibitor (BestBio, Xi’an, China). The homogenate was centrifuged at 1000 g at 4 °C for 5 min. The supernatant was preserved as cytoplasm and placed on ice for 30 min. Then, 1 μL of protease inhibitor and 1 μL of phosphatase inhibitor (BestBio, Xi’an, China) were added to 200 μL of Extract B and 5 μL of Extract C. The mixture is placed on ice for another 30 min and kept as a nucleus. The precipitates were analyzed by standard immunoblotting procedures.

### 2.9. Virus Titer

BHK-21 cells grown in 96-well plates were infected with 10-fold serial dilutions of PRV samples. After 2 h at 37 °C, the culture medium was replaced with fresh DMEM. The plates were incubated for 72–96 h at 37 °C. PRV titers were calculated using the Reed–Muench method.

### 2.10. ICP0 mRNA Detection

PK15 cells were infected with 1 MOI PRV, and then cells were collected at 1 h, 2 h, 3 h, 4 h and 5 h post-infection. Cellular RNA was extracted, and the expression of ICP0 mRNA expression was detected by RT-qPCR. Primers used were as follows: ICP0-qF, GCGACGCTTCGTTTGTGG; ICP0-qR, GGTTCATCCCGTGCTCCTG.

### 2.11. Enzyme-Linked Immunosorbent Assay (ELISA)

IFN-β secretion expression levels in the cell supernatants were detected using a swine IFN-β ELISA kit (Jianglaibio, Shanghai, China), according to the manufacturer’s instructions.

### 2.12. P65 Polyubiquitination Assay

PK15 cells were co-transfected with the Myc-tagged ICP0, FLAG-tagged p65 and Myc-tagged Ub expression vector at a 1:1:1 ratio using the Lipofectamine 3000 transfection method. Protein was extracted 30 h post-transfection. The p65–ubiquitin complexes were immunoprecipitated using anti-Flag antibody and immunoblotted with anti-Myc antibody to detect ubiquitinated proteins.

### 2.13. Statistical Analysis

Measurements were compared using one-way ANOVA. Statistical significance comparisons were calculated using Student’s *t*-test in GraphPad Prism 7.0 software (La Jolla, CA, USA). Unless otherwise stated, data are presented as the mean ± standard deviation (SD) of at least three independent experiments. Asterisks indicate statistically significant differences (*** *p* < 0.001, ** *p* < 0.01 and * *p* < 0.05).

## 3. Results

### 3.1. ICP0 Promotes PRV Replication in PK15 Cells

**Figure 1:**
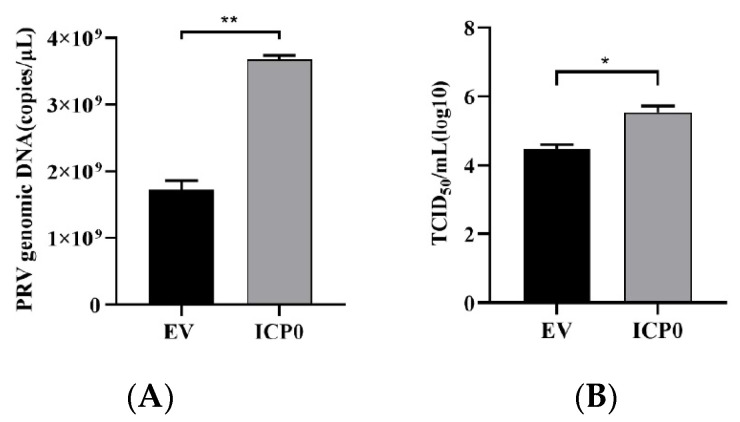
Exogenous expression of ICP0 enhances PRV replication in vitro. PK15 cells were transfected with 1 μg pCMV-Myc plasmid (empty vector, EV) or pCMV-Myc-ICP0 plasmid (ICP0) for 24 h. Then, cells were infected with 0.01 MOI PRV for 24 h before PRV viral copy numbers and titers were measured. Real-time quantitative PCR was used to determine viral copy number (**A**), and TCID50 assay was performed for viral titer detection (Reed–Muench method) (**B**). Data are listed as mean ± SD from three independent experiments. Comparison between two groups was evaluated by unpaired Student’s *t*-test. * *p* < 0.05, ** *p* < 0.01.

To investigate the role of viral protein ICP0 in the process of PRV infection, pCMV-Myc-ICP0 plasmid was transfected into PK15 cells. After 24 h, cells were inoculated with PRV to observe the effect of ICP0 on PRV multiplication. RT-qPCR results show that the amount of viral genomic DNA copies in the ICP0 group grew considerably as compared to the empty vector (EV) group (Figure 1A). Meanwhile, TCID_50_ assay showed the same result: there was a significant upregulation of virus titer in the ICP0 transfection group (Figure 1B). These results suggest that PRV viral protein ICP0 significantly promotes the replication of PRV in PK15 cells.

### 3.2. ICP0 Inhibits the Transcription of Inflammatory Factors

**Figure 2:**
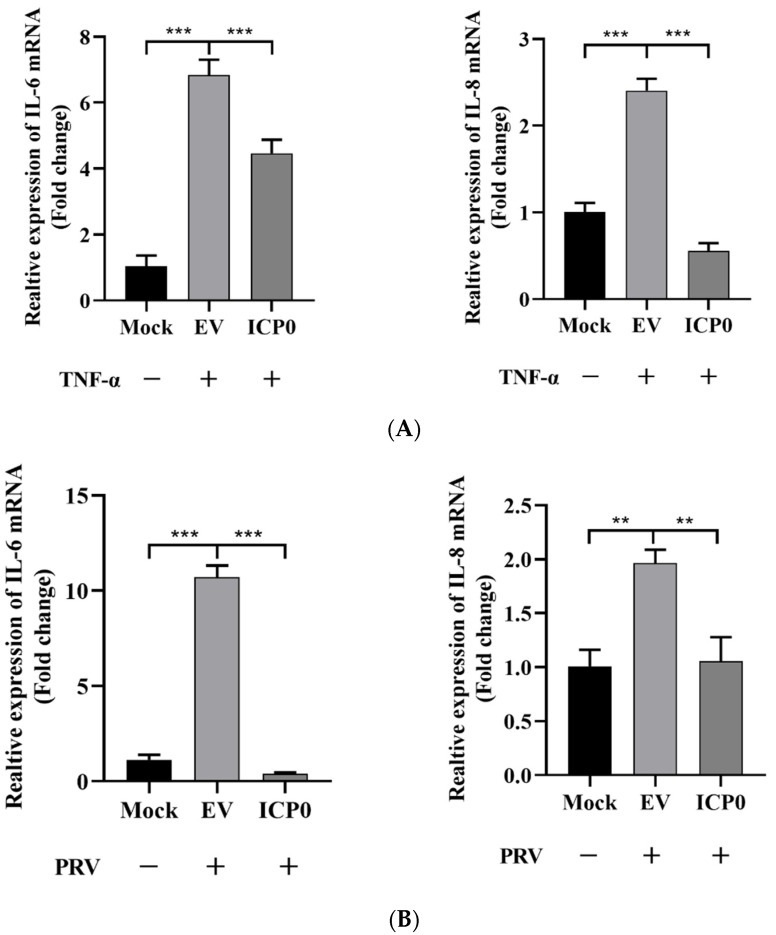
Viral protein ICP0 inhibits IL-6 and IL-8 mRNA transcription. (**A**) PK15 cells were transfected with pCMV-Myc empty vector (EV) (1.0 µg) or pCMV-Myc-ICP0 (1.0 µg) plasmids for 24 h. Then, cells were treated with or without 50 ng/mL recombinant human TNF-α and incubated for an additional 12 h, followed by total RNA extraction. IL-6 and IL-8 mRNA expression levels were measured by RT-qPCR. (**B**) PK15 cells were transfected with pCMV-Myc empty vector (EV) (1.0 µg) or pCMV-Myc-ICP0 (1.0 µg) plasmids for 24 h. Then, cells were infected with 0.01 MOI PRV for another 24 h. Cells were collected for cellular RNA extraction, and RT-qPCR was performed for IL-6 and IL-8 mRNA detection. Data are shown as mean ± SD from three independent experiments. Comparison between two groups was evaluated by unpaired Student′s *t*-test. ** *p* < 0.01, *** *p* < 0.001.

To investigate whether ICP0 is involved in the regulation of inflammatory factor expression, we assessed the effect of ICP0 on IL-6 and IL-8 mRNA transcription by RT-qPCR. TNF-α was used as a stimulatory factor, and PK15 cells were transfected with an ICP0 expression plasmid for 24 h prior to stimulation. Interestingly, TNF-α significantly increased IL-6 and IL-8 mRNA expression, while gene transcription levels were significantly reduced in ICP0 expression cells (Figure 2A). In addition, IL-6 and IL-8 could be efficiently activated during PRV infection; in contrast, in viral protein ICP0 expression groups, this was accompanied by a significant reduction in the transcription of these genes (Figure 2B). Indeed, these results indicated that ICP0 protein dramatically inhibited TNF-α-mediated NF-κB activation.

### 3.3. p65 May Be a Target of Pseudorabies Virus ICP0 Protein

**Figure 3:**
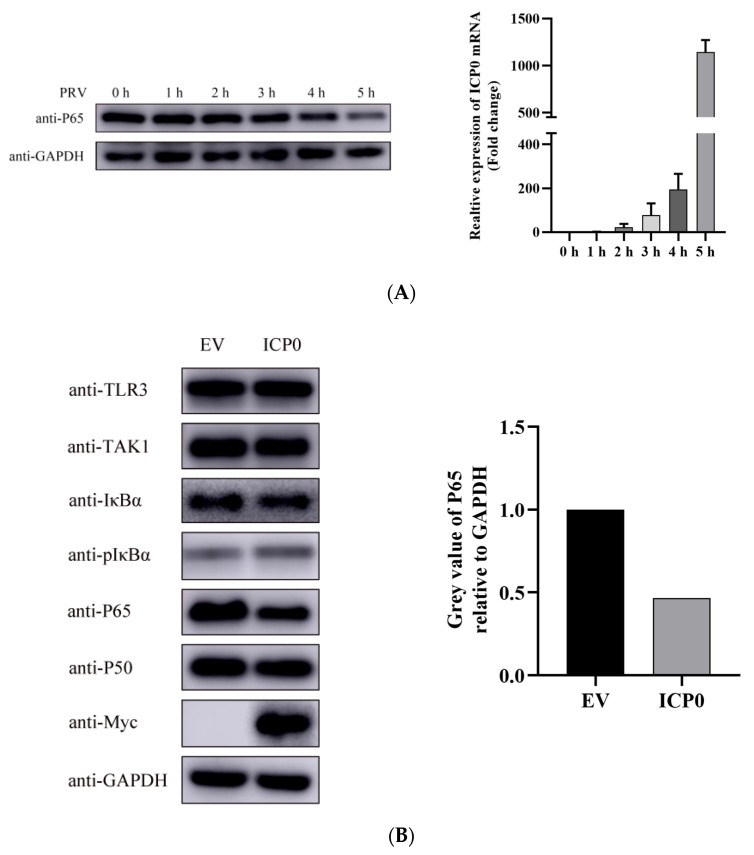

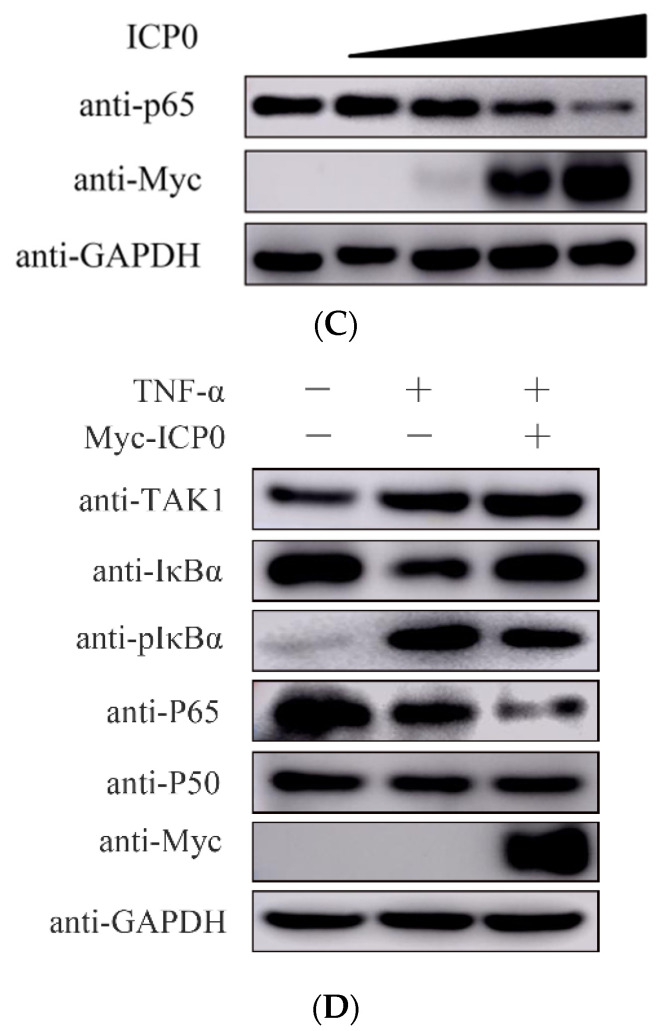
p65 may be a target of pseudorabies virus ICP0 protein. (**A**) PK15 cells were mock infected or infected with 1 MOI PRV for 1 h, 2 h, 3 h, 4 h and 5 h. Then, cells were collected at the indicated time points. Western blot was performed for p65 expression detection (Left). The expression of ICP0 mRNA was detected by RT-qPCR (Right). GAPDH served as loading control. (**B**) PK15 cells were transfected with pCMV-Myc empty vector (EV) (1.0 µg) or Myc-ICP0 (1.0 µg) plasmids for 24 h. Then, cells were harvested and lysed for Western blot detection. Factors included in the NF-κB signaling pathway such as TAK1, IκBα, pIκBα, p50 and p65 were detected. GAPDH served as loading control. The grey value of p65 relative to GAPDH was quantified. (**C**) PK15 cells were transfected with increasing concentrations of expression vectors for ICP0. After 24 h, cells were collected and lysed for p65 detection. GAPDH served as loading control. The grey value of p65 protein expression relative to GAPDH was also quantified. (**D**) PK15 cells were transfected with pCMV-Myc empty vector (EV) (1.0 µg) or Myc-ICP0 (1.0 µg) plasmids for 24 h. Then, cells were treated with or without 50 ng/mL recombinant human TNF-α and incubated for an additional 12 h. Western blot was performed for TAK1, IκBα, pIκBα, p50 and p65 detection with the indicated antibodies. GAPDH served as loading control.

During PRV infection, we found PRV infection induced p65 degradation at 5 h post-infection at 1 MOI infection (Figure 3A left). When infected with a lower MOI (0.01 MOI), the degradation of p65 started at 12 h post-infection (data not shown). This indicated there might be other viral proteins participating in degrading p65 in the late infection of PRV. We also detected ICP0 mRNA expression in 1 MOI PRV infected cells at indicated time points. Results showed that ICP0 mRNA expression was increased with infection time (Figure 3A Right). This means p65 degradation may be associated with ICP0 mRNA transcription in infected cells. In order to determine whether p65 of the NF-κB signaling pathway is ICP0’s molecular target, we overexpressed the viral protein ICP0 in PK15 cells. Western blot results showed that ICP0 was able to block the phosphorylation of IκBα, as well as the degradation of p65 (Figure 3B). Interestingly, the degradation of p65 by ICP0 was shown to proceed in a dose-dependent manner (Figure 3C). Based on the previous results, we can speculate that the degradation of p65 upon PRV infection may be related to viral protein ICP0.

Next, we wanted to verify whether ICP0 played a similar role in the TNF-α-induced NF-κB signaling pathway activation. TNF-α was used as a stimulating factor in ICP0-transfected PK15 cells. The results showed that TNF-α efficiently activated the NF-κB pathway, but ICP0 overexpression significantly inhibited IκBα phosphorylation and degraded p65 (Figure 3D), which is consistent with our previous results. Moreover, we also found when the NF-κB pathway was activated during TNF-α stimulation, ICP0 could inhibit the degradation of IκBα. This result was different from the result shown in Figure 3B, which indicated that ICP0 had no effect on IκBα unless the NF-κB pathway was activated. Based on these results, we inferred that ICP0 may target p65 or its downstream to inhibit NF-κB pathway activation.

### 3.4. ICP0 Interacts with p65 and Degrades p65 through the Proteasome Pathway

**Figure 4:**
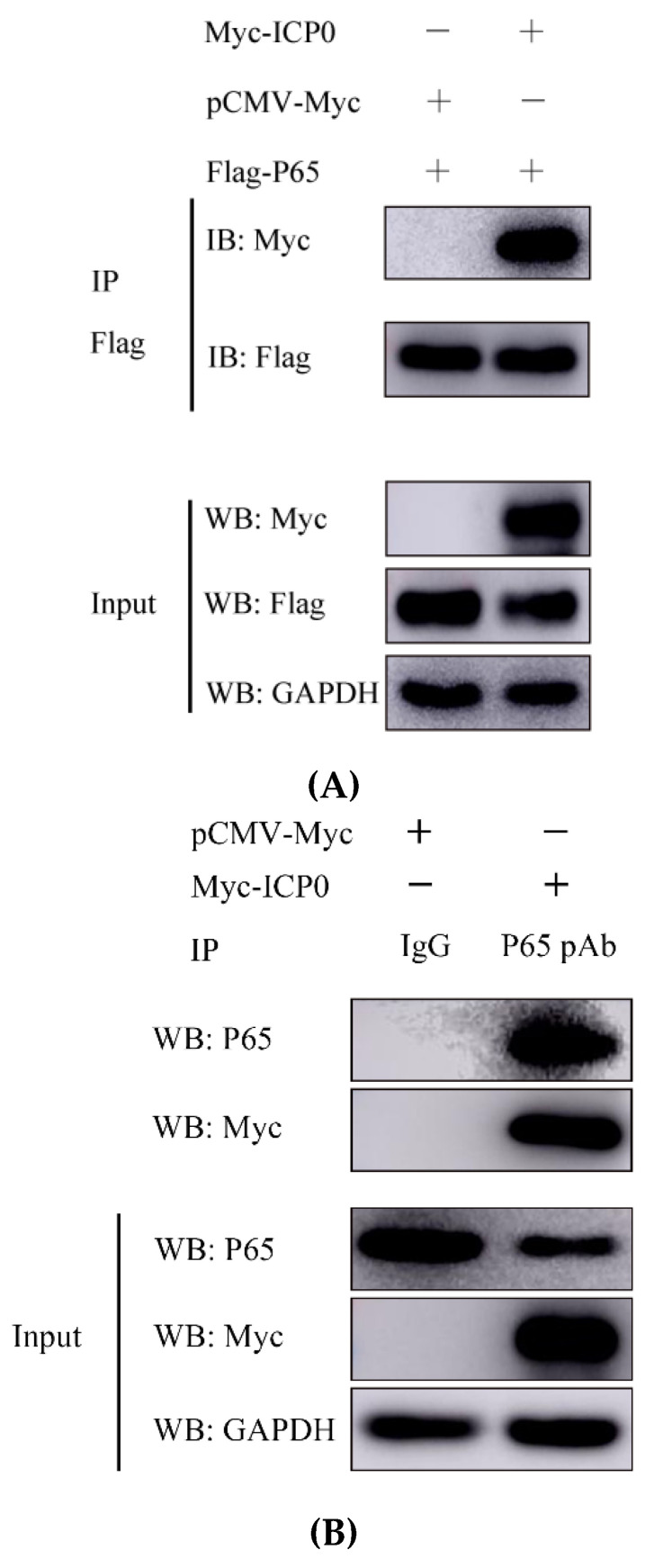

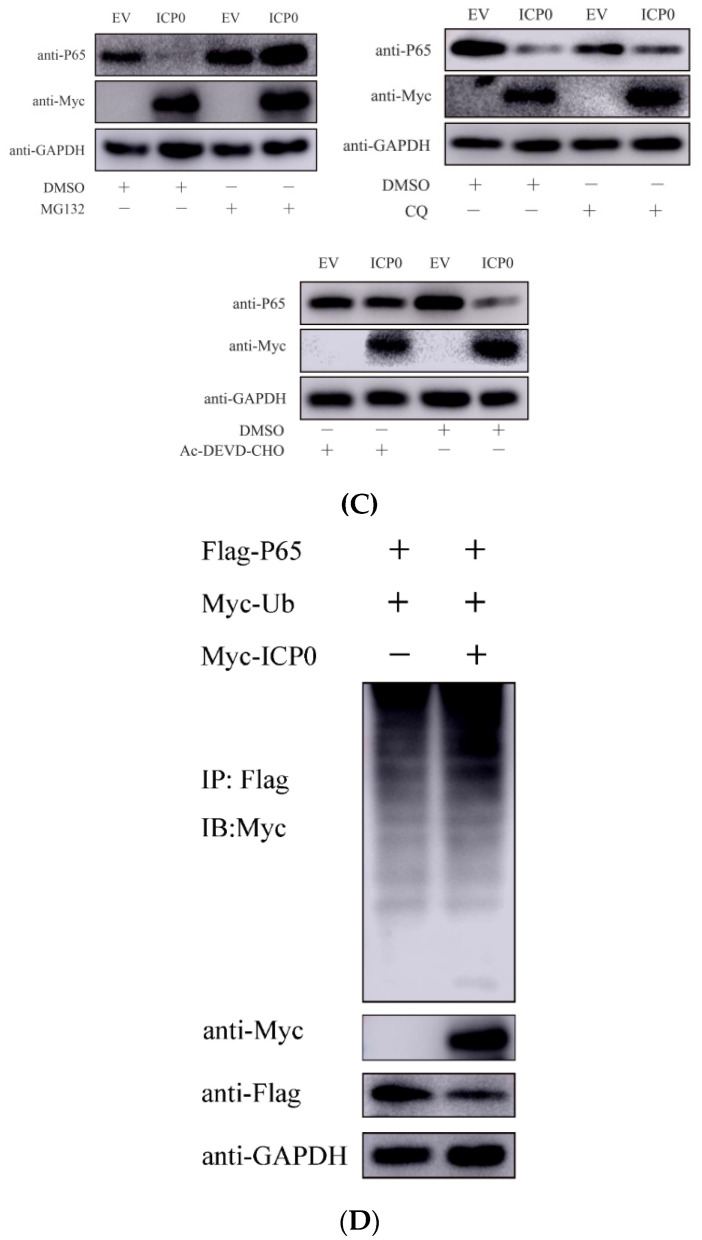
ICP0 targets p65 to inhibit NF-κB signaling. (**A**) PK15 cells were co-transfected with pCMV-Myc empty vector (1.0 µg) or Myc-ICP0 (1.0 µg) plasmids and Flag-p65 (1.0 µg) plasmids for 24 h. The cells were then lysed and immunoprecipitated with an anti-Flag antibody. The whole-cell lysates (input) and immunoprecipitation (IP) complexes were analyzed using an anti-Myc, anti-Flag or anti-GAPDH antibody by Western blotting. (**B**) PK15 cells were transfected with pCMV-Myc (1.0 µg) or Myc-ICP0 (1.0 µg) plasmids for 24 h. The cells were lysed and immunoprecipitated with an anti-p65 antibody. The input and IP complexes were analyzed by Western blotting using anti-p65, anti-Myc or anti-GAPDH antibodies. (**C**) PK15 cells were transfected with Myc-ICP0 (1.0 µg) or pCMV-Myc empty vector (EV) (1.0 µg) for 24 h, then treated with proteasomal inhibitor MG132 (7.5 µM), lysosome inhibitor CQ (50 µM) or caspase 3 inhibitor Ac-DEVD-CHO (50 µM) for 12 h. DMSO-treated cells served as vehicle control. Then, cells were collected and immunoblotted for p65 and Myc-tagged ICP0. GAPDH served as loading control. (**D**) PK15 cells were co-transfected with the Myc-tagged ICP0, FLAG-tagged p65 and Myc-tagged Ub 30 h post-transfection. The p65–ubiquitin complexes were immunoprecipitated using anti-Flag antibody and immunoblotted with anti-Myc antibody to detect ubiquitinated proteins.

PRV ICP0 exhibited a remarkable inhibitory effect on p65 protein, suggesting it could target p65. To investigate whether there is an interaction between ICP0 and p65, PK15 cells were co-transfected with Flag-p65 and Myc-ICP0 plasmids for 30 h. Cells were collected and a co-immunoprecipitation assay was carried out. The results showed that p65 co-precipitated with ICP0 protein (Figure 4A), suggesting that there is a direct interaction between p65 and ICP0. We have further demonstrated this interaction under physiological conditions. Myc-ICP0 plasmid was transfected into PK15 cells and verified using immunoprecipitation. Interestingly, we obtained similar results (Figure 4B), which would further certify an interaction between p65 and ICP0.

Next, to identify the pathway by which ICP0 achieves the degradation of p65, we transfected the Myc-ICP0 plasmid into PK15 cells and treated the cells with MG132 (ubiquitin–proteasome inhibitor), chloroquine (CQ, lysosome pathway inhibitor) or Ac-DEVD-CHO (caspase-3 inhibitor). Western blot results showed that MG132 prevented the degradation of p65 by ICP0 but not by CQ and caspase-3 inhibitor Ac-DEVD-CHO (Figure 4C). It is suggested that the degradation of p65 by ICP0 is achieved through the ubiquitin–proteasome pathway. Proteins degraded via the proteasome pathway must be ubiquitinated first. The above data indicated that ICP0 interacted well with p65; we next examined whether ICP0 affects p65 ubiquitination. The ubiquitination assay showed that ICP0 could increase p65 polyubiquitination (Figure 4D).

### 3.5. ICP0 Protein Suppresses p65 Phosphorylation

**Figure 5:**
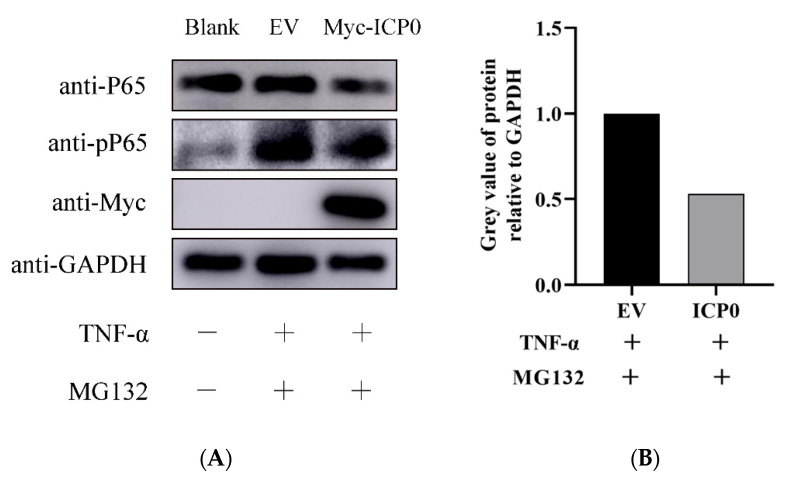
ICP0 protein suppresses p65 phosphorylation. (**A**)PK15 cells were transfected with empty vector (EV) (1.0 µg) or Myc-ICP0 (1.0 µg) plasmids for 24 h and then stimulated with TNF-α (50 µg/mL) for 12 h. Cells were treated with 7.5 µM MG132 for another 12 h before collection. p65, phosphorylated p65 (pP65) and ICP0 protein (Myc) expression were detected by immune blotting. GAPDH served as loading control. (**B**) The grey value of p65 phosphorylation relative to GAPDH was quantified.

The classical hallmark of NF-κB activation is the degradation of IκBα, releasing two subunits, namely p50 and p65, which then undergo phosphorylation and ubiquitination modifications in the nucleus. ICP0 affects the NF-κB signaling pathway by targeting p65; therefore, it was necessary to verify the effect of ICP0 on p65 phosphorylation. Empty vector (EV) and Myc-ICP0 plasmids were transfected into PK15 cells respectively. Then, cells were treated with MG132 and TNF-α. The results showed that TNF-α induced significant phosphorylation of p65. Meanwhile, in the ICP0 transfection group, the phosphorylation level of p65 was effectively inhibited (Figure 5). These results indicated that ICP0 abrogated p65 phosphorylation.

### 3.6. ICP0 Protein Blocks p65 Nuclear Translocation

**Figure 6:**
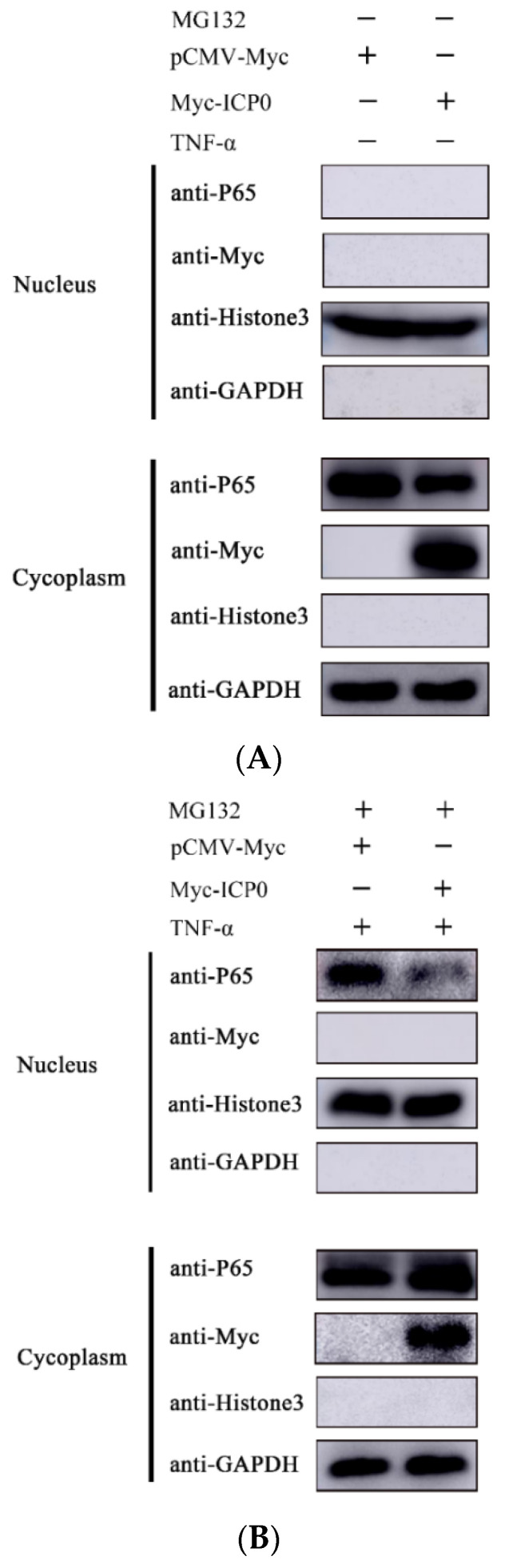
ICP0 protein blocks p65 nuclear translocation. (**A**) PK15 cells were transfected withpCMV-Myc (1.0 µg) or Myc-ICP0 (1.0 µg) for 36 h. Cells were collected without MG132 and TNF-α treatment. Cytoplasmic and nuclear proteins were extracted and subjected to Western blot analysis.(**B**) TNF-α (50 µg/mL) was added into PK15 cells in the presence of either pCMV-Myc (1.0 µg) or Myc-ICP0 (1.0 µg) for 24 h. Before being collected, cells were treated with MG132 and TNF-α for 12 h. Then, cytoplasmic and nuclear proteins were extracted and subjected to Western blot analysis. Expression of p65 and Myc-tagged ICP0 was detected with specific antibodies. Histone 3 was used as a nuclear protein marker. GAPDH served as loading control.

The phosphorylation of p65 causes its nuclear translocation. To verify whether ICP0 affects the nuclear translocation process of p65, we transfected an ICP0 expression plasmid into PK15 cells and examined the distribution of p65 in the cytoplasm and nucleus by nucleoplasmic separation assay. Compared to the empty vector transfection group, in the ICP0 transfection group, most of the p65 remained in the cytoplasm and only a small amount entered the nucleus (Figure 6). The above results suggest that ICP0 inhibits the TNF-α-induced nuclear translocation process of p65, thereby limiting the activation of the NF-κB signaling pathway.

### 3.7. ICP0 Protein Promotes PRV Proliferation via Decreasing IFN-β Production

**Figure 7:**
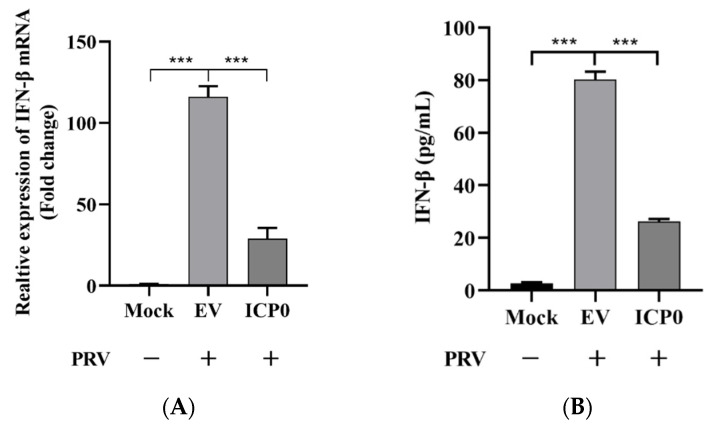
ICP0 protein promotes PRV proliferation via decreasing IFN-β production. (**A**) PK15 cells were transfected with pCMV-Myc and Myc-ICP0 plasmids. Then, cells were infected with PRV. Cellular RNA was extracted, and RT-qPCR was performed to detect IFN-β mRNA transcription. (**B**) PK15 cells were transfected with pCMV-Myc and Myc-ICP0 plasmids. Then, cells were infected with PRV. Cell culture supernatant was collected. The secretion expression of IFN-β was detected using ELISA. Data are shown as mean ± SD from three independent experiments. Comparison between two groups was evaluated by unpaired Student’s *t*-test. *** *p* < 0.001.

In Figure 1, we demonstrate that ICP0 expression enhances PRV replication in PK15 cells, but it is unclear whether this observation is due to the attenuation of cytokine production via NF-κB suppression shown in the above results. As it is well known that NF-κB regulates IFN-β production, we then detected IFN-β expression in empty-vector- or ICP0-transfected cells during PRV infection. Results indicated that ICP0 could abrogate IFN-β mRNA transcription compared to the empty vector group in the condition of viral infection (Figure 7A). ELISA was also used to quantify IFN-β secretion expression. As shown in Figure 7B, ICP0 significantly inhibited antiviral factor IFN-β production. These results demonstrated that ICP0 overexpression could block IFN-β production. Furthermore, the increase in PRV proliferation caused by ICP0 was related to the decrease in antiviral factor IFN-β production.

## 4. Discussion

The innate immune response of the host is thought to play an important role in resistance to viral infection. Activation of NF-κB as a strategy that protects the host against viral pathogens plays a vital role in the regulation of the intrinsic immune response [23,24]. Therefore, many viruses have evolved different strategies to modulate NF-κB activation and thus evade the host immune response [25,26,27]. In this study, we have demonstrated that the PRV-encoded immediate early protein ICP0 inhibits the TNF-α-mediated NF-κB signaling pathway activation.

PRV is a swine alphaherpesvirus closely related to the human herpes simplex virus type 1 (HSV-1). PRV infects a broad host range of mammals. PRV infection primarily causes an acute lytic infection in the adult pig, its natural host, characterized by respiratory distress and reproductive failure while resulting in neurological symptoms and high mortality in newborn piglets and non-natural hosts [28]. Although hosts have evolved powerful innate immune mechanisms in response to virus invasion, PRV has evolved strategies to hijack host immune responses for viral replication and the establishment of persistent infection.

In the present study, the PRV-encoded ICP0 protein has been verified to have the ability to prevent TNF-α-stimulated NF-κB signaling pathway activation (Figure 2 and Figure 3). Given that the innate immune response is the first line of host antiviral systems, these results indicate that ICP0 plays an important role in PRV immune evasion of the NF-κB signaling transduction pathway. The NF-κB pathway controls the transcription of many immune molecules required to initiate an immune response to foreign pathogens; as a result, disruption of the NF-κB pathway is likely to inhibit the immune response capacity of the host cell.

PRV uses numerous viral proteins to antagonize the host innate immune system. Previous studies showed that PRV UL24 had an inhibitory role in the NF-κB pathway [21]. In this study, we proved that PRV ICP0 had a similar effect to UL24 in the inhibition of the NF-κB pathway. Using Western blot analysis, we identified p65 as a target of the PRV ICP0 protein, through which it inhibits TNF-α-mediated activation of the NF-κB signaling pathway (Figure 3).

p65 is a key regulator of the NF-κB pathway [29]; it can be phosphorylated by cellular and viral proteins, contributing to the activation or inhibition of the transcriptional activity of p65 and, as a result, leading to an increase or decrease in the production of inflammatory factors.

Here, we found that the PRV ICP0 protein antagonized the NF-κB pathway by targeting p65 and inhibited p65 phosphorylation and nuclear translocation (Figure 5 and Figure 6). Moreover, there was a direct interaction between ICP0 and p65, and endogenous p65 expression was also degraded by ICP0 through the proteasomal pathway (Figure 4).

As there is a direct interaction between ICP0 and p65 (Figure 4), and endogenous p65 levels are affected by ICP0 (Figure 3), we can infer that the nucleus p65 decrease in non-TNF-α/MG132-treated cells is related to the degradation effect of ICP0 on p65. In contrast, in TNF-α/MG132-treated cells, compared with the empty-vector-transfected group, in the ICP0-transfected group, most of the p65 protein remained in the cytoplasm, and only a small amount of p65 entered the nucleus (Figure 6). These results further confirmed that ICP0 could inhibit the nuclear translocation of ICP0. In addition, results in Figure 3 and Figure 5 also indicated that ICP0 could inhibit the phosphorylation level of IκBα and p65 when cells were treated with TNF-α or TNF-α combined with MG132. So, the different results in Figure 6A,B may be due to the inhibitory effect of ICP0 on phosphorylation of IκBα or p65.

In a related study on herpes simplex virus 1 study, scientists found that its ICP0 protein inhibits TNF-α-induced NF-κB activation by interacting with p65 and p50. HSV-1 ICP0 also degraded p50 via its E3 ubiquitin ligase activity. Our results showed that PRV ICP0 had no effect on p50. Although both HSV-1 and PRV belong to Herpesviridae, and ICP0 is a relatively conserved viral protein, the mechanisms by which it plays a role in different viral infections may be different. As mentioned in this study, the ICP0 proteins of the two viruses, which both affect the NF-κB signaling pathway, have different target molecules. This difference may be related to the infectious properties of the viruses, and it also greatly enriches our understanding of the role of ICP0 protein in the natural immune response.

**Figure 8:**
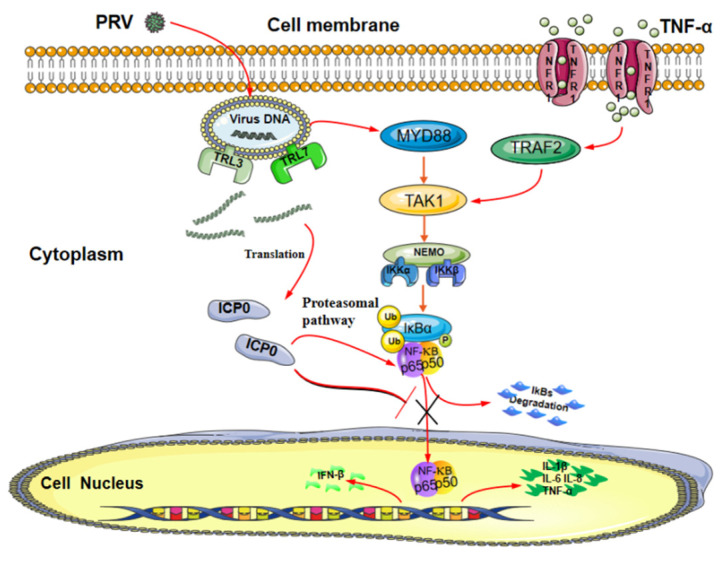
Model of ICP0 interfering with the NF-κB signaling pathway. The PRV protein ICP0 blocks the degradation of IκBα and inhibits its phosphorylation. Meanwhile, ICP0 interacts with p65 and degrades p65 protein expression via the proteasome pathway. In addition, ICP0 inhibits p65 phosphorylation and prevents its nuclear translocation, thereby negatively regulating the NF-κB signaling pathway.

In summary, our data demonstrate a possible mechanism by which ICP0 abolishes the NF-κB signaling pathway (Figure 8). ICP0 inhibits NF-κB activation by targeting p65, and there is a direct interaction between ICP0 and p65. In addition, ICP0 decreases the expression of p65 and blocks the activation of the NF-κB pathway by inhibiting phosphorylation and nuclear translocation of p65. These findings suggest that pseudorabies virus ICP0 can inhibit the TNF-α-mediated NF-κB signaling pathway and provide new insights into the innate immune evasion of the pseudorabies virus.

## Data Availability

All available data are presented in the article.
